# Brazilian guidelines for the pharmacological treatment of pulmonary hypertension. Official document of the Brazilian Thoracic Society based on the Grading of Recommendations Assessment, Development, and Evaluation methodology

**DOI:** 10.36416/1806-3756/e20250528

**Published:** 2026-08-13

**Authors:** Veronica Moreira Amado, Mônica Corso Pereira, Ricardo Amorim Correa, Frederico A F Thadeu Campos, Caio Fernandes, Eloara V M Ferreira, Marcelo B Gazzana, Marcelo Jorge Jacó Rocha, Carlos Jardim, Jose Leonidas Alves-Jr, Rudolf K F Oliveira, Virginia Pacheco, William Salibe-Filho, Suzana Tanni, Daniel Waetge, Jaquelina S Ota-Arakaki, Rogerio Souza

**Affiliations:** 1. Faculdade de Medicina, Universidade de Brasília - UNB - Brasília (DF) Brasil.; 2. Serviço de Pneumologia, Departamento de Clínica Médica, Faculdade de Ciências Médicas, Universidade Estadual de Campinas - UNICAMP - Campinas (SP) Brasil.; 3. Medicina Interna/Divisão de Pneumologia, Faculdade de Medicina, Universidade Federal de Minas Gerais, Belo Horizonte (MG) Brasil.; 4. Instituto Pequenas Missionárias de Maria Imaculada/Hospital Madre Teresa, Belo Horizonte (MG) Brasil.; 5. Unidade de Circulação Pulmonar, Instituto do Coração, Hospital das Clínicas, Faculdade de Medicina, Universidade de São Paulo, São Paulo (SP) Brasil.; 6. Divisão de Doenças Respiratórias, Hospital São Paulo, Universidade Federal de São Paulo - Unifesp - São Paulo (SP) Brasil.; 7. Serviço de Pneumologia, Hospital de Clínicas de Porto Alegre, Porto Alegre (RS) Brasil.; 8. Hospital de Messejana Dr Carlos Alberto Studart Gomes, Fortaleza (CE) Brasil.; 9. Faculdade de Medicina de Botucatu, Universidade Estadual de São Paulo - UNESP - Botucatu (SP) Brasil.; 10. Departamento de Clínica Médica, Divisão de Pneumologia, Faculdade de Medicina, Universidade Federal do Rio de Janeiro, Rio de Janeiro (RJ) Brasil.

**Keywords:** Pulmonary arterial hypertension/drug therapy, Hypertension, pulmonary/classification, Hypertension, pulmonary/drug therapy, Hipertensão arterial pulmonar, Hipertensão pulmonar, Tratamento

## Abstract

Pulmonary hypertension (PH) is a complex and heterogeneous clinical condition associated with high morbidity and mortality and can occur in the context of various diseases. It is characterized by increased pulmonary arterial pressure and progressive right ventricular overload. The current classification of the different forms of PH presentation comprises five groups, based on pathophysiological similarities and available therapeutic options. In recent decades, significant advances in pathophysiological understanding and the development of specific therapies have significantly modified the prognosis of patients with PH. This guideline aims to define updated Brazilian recommendations for the treatment of PH, with an emphasis on pulmonary arterial hypertension, considering the available scientific evidence and the particularities of the Brazilian health system. Four structured questions in the PICO format (patients, intervention, comparator group, and outcome) were developed by a multidisciplinary panel of Brazilian experts. Systematic literature reviews were conducted for each question, based on randomized clinical trials. Meta-analyses were conducted where methodologically feasible. The quality of evidence and the strength of recommendations were defined according to the Grading of Recommendations Assessment, Development and Evaluation (GRADE) system. The guideline was complemented by a comprehensive narrative review of the literature on clinical management of patients with PH, especially patients with pulmonary arterial hypertension, addressing general measures, specific therapeutic strategies, risk stratification, and treatment algorithm, always based on the best available scientific evidence and with the aim of contributing to the improvement of care for patients with PH in Brazil.

## RESUMO

A hipertensão pulmonar (HP) é uma condição clínica complexa e heterogênea, associada a elevada morbimortalidade e que pode ocorrer no contexto de diversas doenças. Caracteriza-se pelo aumento da pressão arterial pulmonar e pela progressiva sobrecarga ventricular direita. A classificação atual das diferentes formas de apresentação da HP compreende cinco grupos, definidos a partir de semelhanças fisiopatológicas e das opções terapêuticas disponíveis. Nas últimas décadas, avanços significativos no entendimento fisiopatológico e no desenvolvimento de terapias específicas modificaram de forma relevante o prognóstico dos pacientes com HP. Esta diretriz tem como objetivo definir recomendações brasileiras atualizadas para o tratamento da hipertensão pulmonar, com ênfase na hipertensão arterial pulmonar, considerando as evidências científicas disponíveis e as particularidades do sistema de saúde brasileiro. Foram elaboradas quatro perguntas estruturadas no formato PICO [pacientes, intervenção, grupo comparador e outcome (desfecho)] por um painel multidisciplinar de especialistas brasileiros. Para cada questão, foram realizadas revisões sistemáticas da literatura a partir de ensaios clínicos randomizados, com metanálise quando metodologicamente viável. A qualidade das evidências e a força das recomendações foram definidas de acordo com o sistema GRADE (Grading of Recommendations Assessment, Development and Evaluation). O texto da diretriz foi complementado pela revisão narrativa abrangente da literatura sobre o manejo clínico dos pacientes com HP, em especial, pacientes com hipertensão arterial pulmonar, abordando medidas gerais, estratégias terapêuticas específicas, estratificação de risco e algoritmo de tratamento, sempre com base nas melhores evidências científicas disponíveis e com o objetivo de contribuir para a qualificação da assistência aos pacientes com hipertensão pulmonar no Brasil.

## INTRODUCTION

Pulmonary hypertension (PH) is a clinical condition that can be associated with various cardiopulmonary, autoimmune, infectious, hematological, and inflammatory diseases. The various forms of PH presentation are categorized into five groups, according to the current classification, considering pathophysiological similarities and available treatments (Chart 1).^1^ The definition of PH is based on hemodynamic criteria obtained invasively through right heart catheterization (RHC). Thus, PH is considered in the presence of mean arterial pressure (mPAP) greater than 20 mmHg. In pre-capillary PH, pulmonary vascular resistance (PVR) is greater than 2 WU and pulmonary artery occlusion pressure (PAOP) is less than or equal to 15 mmHg. In post-capillary PH, PVR is less than or equal to 2 WU and PAOP is greater than 15 mmHg. Combined PH, with pre- and post-capillary components, is characterized by a PAOP greater than 15 mmHg and a PVR greater than 2 WU.^1^ Unclassifiable PH stands out, in which there is an elevation in mPAP, but PVR < 2 WU and PAOP ≤ 15 mmHg, generally associated with high cardiac output conditions.^2^

**Chart 1:** Classification of pulmonary hypertension.

1. Pulmonary arterial hypertension (PAH) 1.1. Idiopathic 1.1.1. Long-term responders to calcium channel blockers 1.2. Hereditary 1.3. Associated with drugs and toxins 1.4. Associated with: 1.4.1. Connective tissue disease 1.4.2. Human immunodeficiency virus (HIV) infection 1.4.3. Portal hypertension 1.4.4. Congenital heart diseases 1.4.5. Schistosomiasis 1.5. PAH with characteristics of venous/capillary involvement (veno-occlusive PH/pulmonary capillary hemangiomatosis) 1.6. Persistent PH of the newborn
2. PH due to left heart disease 2.1. Heart failure: 2.1.1. With preserved ejection fraction 2.1.2. With reduced or slightly reduced ejection fraction 2.1.3. Cardiomyopathies with specific etiologies 2.2. Heart valve disease 2.2.1. Aortic valve disease 2.2.2. Mitral valve disease 2.2.3. Mixed valve disease 2.3. Congenital/acquired cardiovascular conditions leading to post-capillary PH
3.1. COPD and/or emphysema 3.2. Interstitial lung disease 3.3. Combined pulmonary fibrosis-emphysema syndrome 3.4. Other parenchymal lung diseases 3.5. Nonparenchymal restrictive diseases 3.5.1 Hypoventilation syndromes 3.5.2 Pneumectomy 3.6. Lung developmental diseases
4. PH with pulmonary artery obstructions 4.1. Chronic thromboembolic PH (CTEPH) 4.2. Other pulmonary artery obstructions: angiosarcoma, other intravascular tumors, arteritis, congenital stenosis of the pulmonary arteries, parasites (hydatidosis)
5. PH with uncertain and/or multifactorial mechanisms 5.1. Hematological diseases: chronic hemolytic anemia, myeloproliferative diseases, splenectomy 5.2. Systemic diseases: sarcoidosis, pulmonary Langerhans cell histiocytosis, neurofibromatosis type I 5.3. Metabolic diseases: glycogen storage disease, Gaucher disease, thyroid disorders 5.4. Chronic renal failure (with or without hemodialysis) 5.5. Lung tumor thrombotic microangiopathy 5.6. Fibrosing mediastinitis 5.7. Complex congenital heart diseases

The diagnostic assessment of PH is extensive, complex, and fundamental for the correct classification of the disease and for the appropriate therapeutic planning of patients (Chart 1).^2^

In this context, patients with suspected pulmonary arterial hypertension (PAH) (Group 1) and chronic thromboembolic pulmonary hypertension (CTEPH; Group 4) should be followed up in centers specializing in pulmonary circulation diseases. Since the last guideline for the management of PH, published in 2005 by the Brazilian Journal of Pulmonology, there has been great progress in the pathophysiological understanding and in the development of new drugs for the treatment of PH, especially for groups 1 and 4.^3^ This guideline aims to update knowledge regarding the treatment of PH, with a greater focus on Group 1, and to discuss treatment strategies considering the Brazilian scenario. It is expected, therefore, that it will contribute significantly to discussions with public and private managers regarding health care strategies for these patients.

## METHODOLOGY

The steps in developing the guidelines followed the model approved and proposed by the Brazilian Thoracic Society. The questions regarding the pharmacological treatment of patients diagnosed with PH were formulated by specialists, using the PICO [patients, intervention, comparator group, and outcome] format. The outcomes of interest for each question were defined a priori (supplementary material; Table S1).

The search was conducted systematically and the team of experts in evidence-based medicine selected the articles to be included in the systematic review (Tables S2.1-S2.4). The reasons for inclusion or exclusion were recorded and are presented in accordance with the recommendations of the Preferred Reporting Items for Systematic Reviews and Meta-Analyses (PRISMA; Tables S3.1-S3.4).^4^ The results of studies identified with the same intervention were grouped. For each of the four PICO questions, the quality of evidence from the studies included in the systematic review was assessed in accordance with the Grading of Recommendations Assessment, Development, and Evaluation (GRADE) methodology.^5^

The quality of evidence depends on the design and implementation of the studies, as well as risks of bias, which can be classified as high, moderate, low, or very low (Chart 2). Quality can be reduced by one or two levels when risk of bias, indirect evidence, inconsistency, imprecision, or publication bias are identified. On the other hand, quality can be higher when the intervention is strongly associated with the effect, without identification of plausible confounding factors, when there is evidence of dose-response, or when plausible confounding factors are reducing the effect size (Chart 3).^6^ ^-^ ^8^ This step was performed by the methodologists separately, and then the agreement of the assessments was evaluated until they reached a consensus.

**Chart 2:** Interpretation of the quality of evidence according to the Grading of Recommendations Assessment, Development and Evaluation (GRADE) system.

Quality of evidence	Implications	Examples
High (⊕⊕⊕⊕)	It is unlikely that future research will alter confidence in the estimated effect; we are confident that we can expect a very similar effect in the population for which the recommendation is intended.	Randomized trials without serious limitations. Well-executed observational studies with a very large effect.
Moderate (⊕⊕⊕O)	It is likely that future research will have a significant impact on confidence in the estimated effect and may alter this estimate.	Randomized trials with serious limitations. Well-executed observational studies with a large effect.
Low (⊕⊕OO)	It is likely that future research will have a significant impact on confidence in the estimated effect and will probably alter this estimate.	Randomized trials with very serious limitations. Observational studies without special strengths or significant limitations.
Very low (⊕OOO)	Any estimate of an effect is highly uncertain.	Randomized trials with very serious limitations and inconsistent results. Observational studies with serious limitations. Non-systematic clinical observations (e.g., case series or case reports).

**Chart 3:** Factors that can affect the quality of evidence.

Quality of evidence	Situations in which grading may be reduced	Situations in which the grading may be elevated
• High • Moderate • Low • Very low	• Risk of bias • Indirect evidence • Inconsistency • Imprecision • Publication bias	• Strong association, without plausible confounders • Evidence of dose-response • Plausible confounders reducing the effect

The coordinating committee met with members of the expert committee to review all charts containing evidence summaries (Tables S4.1-S4.4). Recommendations for each question were classified as strong or conditional, according to the certainty about the strength and quality of evidence, among other factors. Similar to the GRADE methodology, we used the term “recommended” for strong recommendations and “suggested” for conditional recommendations. The GRADE tool known as the “evidence to decision framework” (EtD) was used to ensure that the following factors were considered: balance between desirable and undesirable effects of the intervention, degree of certainty (based on the quality of existing evidence), patient values and preferences, implications for resource use, and feasibility of implementation (Tables S5.1-S5.4).^7^ ^,^ ^8^ In situations when there was no consensus, votes were taken and the results were recorded.

In addition to the four PICO questions that followed the GRADE methodology, the text of the guidelines was supplemented by an extensive literature review, prepared by the PH expert group. In this case, the recommendations followed the model described in the supplementary material (Tables S6 and S7).

## PAH

PAH is a condition characterized by pulmonary vascular remodeling, predominantly in the arteriolar territory, which leads to lumen reduction and wall thickening, with consequent increase in resistance and reduction in pulmonary vascular compliance. The increase in PAP is a consequence of increased right ventricular (RV) afterload, which initially maintains coupling with the pulmonary circulation, but, with the progressive increase in PVR, evolves into heart failure and premature death. The therapeutic management of PAH involves implementing general measures and a combination of therapies that act directly on the pulmonary circulation, providing vasodilatory and antiproliferative effects.

## GENERAL MEASURES

General measures help improve symptoms, prevent complications, and promote a better quality of life for patients with PAH, and include:

### Psychological support

Psychological support should be indicated for patients with PAH, who frequently face emotional, socioeconomic, and physical challenges related to the chronic, limiting, and progressive disease (I-C).^9^

### Home oxygen therapy

The indication for oxygen therapy in PH is controversial, since the evidence provided by the small number of studies is limited. Therefore, it is suggested that supplemental oxygen therapy be prescribed for patients with PaO_2_ < 60 mmHg (IIa-C).^10^

### Pulmonary rehabilitation

Supervised physical exercise, as part of a cardiopulmonary rehabilitation program for patients with PAH, without evidence of hemodynamic deterioration, has been shown to significantly improve functional capacity [six-minute walk distance (6MWD) and maximum oxygen consumption (VO_2_ peak) in the cardiopulmonary test], quality of life, and dyspnea, assessed by the Functional Class/World Health Organization (FC-WHO). Therefore, supervised cardiopulmonary rehabilitation should be incorporated into the treatment of patients with PAH after clinical stabilization with the best available pharmacological regimen (I-A).^11^ ^-^ ^13^

### Vaccination

Infections are a major cause of decompensation in patients with PAH, as they can trigger right-sided heart failure and worsen hemodynamics status. It is essential to keep the vaccination schedule up to date, with special attention to annual influenza and SARS-CoV-2 vaccination, as well as immunization against pneumococcus (I-C).^14^ ^,^ ^15^ Recently, evidence has emerged for a respiratory syncytial virus vaccine for patients with severe heart and lung disease over 60 years of age, so such a vaccine may be considered.^16^

### Pregnancy, birth control, and hormone replacement therapy

In recent years, there has been a significant reduction in reported maternal-fetal mortality in case series of pregnant women with PAH, particularly in developed countries where intravenous prostacyclin analogs are available.^17^ However, in developing countries, maternal-fetal mortality rates of up to 50% have been reported, so pregnancy should be contraindicated in patients with PAH. Nevertheless, pregnancy may be considered in specific cases, such as in long-term responders to calcium channel blockers (CCBs) or well-controlled patients at low risk (normal or near-normal hemodynamics), provided that access to close monitoring in specialized centers is ensured throughout the gestational period. Early termination of pregnancy should be considered in cases of high risk of maternal mortality.^18^ If the decision is made to continue the pregnancy, discontinuation of endothelin receptor antagonists, riociguat, and selexipag is indicated.

Effective contraceptive methods should be prescribed for all women of childbearing age with PAH, with preference given to long-acting hormonal contraceptives such as levonorgestrel intrauterine devices (IUDs) or progestin implants, especially in patients using sildenafil or bosentan, due to the possibility of drug interactions and reduced effectiveness of oral contraceptives. IUD insertion should be performed in a hospital setting with adequate analgesia to avoid hemodynamic complications. Estrogens should not be prescribed due to their risk of thrombosis. Contraceptive counseling is an essential part of the therapeutic plan for women with PAH and should be maintained continuously and consistently (I-C).^18^

### Elective surgeries

Patients with PAH undergoing surgical procedures have a higher risk of hemodynamic complications. The risk-benefit ratio of the proposed procedure should be weighed up. Anesthetic strategies, mechanical ventilation, and the use of vasopressors should be defined by a multidisciplinary team with experience in the management of patients with PH and RV dysfunction (IIa-C).^19^

### Air travel

For air travel, it is suggested that patients in functional classes III and IV and/or those with SpO_2_ ≤ 92% use supplemental O_2_, given the lower pressure in the aircraft cabin (IIa-C).^10^ ^,^ ^15^

### Genetic counseling

Genetic testing and counseling for patients with PAH and their families are not routinely performed in Brazil. Identification of the *BMPR2* gene mutation can assist in prenatal risk assessment, pre-implantation genetic diagnosis, guidance on the use of donated gametes, or suggestions for adoption. However, this technology is not available in clinical practice in our country.^20^

### Diuretics

In PAH, right heart failure is associated with systemic fluid retention, reduced renal blood flow, and activation of the renin-angiotensin-aldosterone system. Increased right chambers filling pressures and systemic venous pressure can raise renal interstitial and tubular hydrostatic pressure, which may compromise the glomerular filtration rate. In this situation, the use of diuretics is indicated for the management of fluid overload, especially in cases of peripheral edema and ascites.

The three main classes of diuretics-loop diuretics, mineralocorticoid receptor antagonists and, in selected cases, thiazides-can be used as monotherapy or in combination. Drugs such as furosemide and spironolactone are widely used, requiring individual dose adjustments and rigorous monitoring of renal function, serum electrolytes, and body weight (-IC).^21^ ^,^ ^22^

### Use of oral anticoagulants

The use of vitamin K antagonists or direct oral anticoagulants may be considered in selected cases of PAH and is recommended in all cases of CTEPH. In the case of patients with PAH associated with connective tissue diseases, the use of these agents, not only demonstrated no benefits, but they also may cause harm, and their routine use is not recommended (IIb-C).^23^ ^,^ ^24^

## SPECIFIC TREATMENT

Since the first molecule was identified as an effective treatment for PAH,^25^ many clinical studies have been conducted, focusing on three classic cell signaling pathways involved in the regulation of vascular tone and pulmonary arterial remodeling. The available molecules acting on these pathways act on endothelial dysfunction and vascular remodeling by inhibiting the endothelin pathway and stimulating the prostacyclin (PGI2) and nitric oxide (NO) pathways. More recently, a fourth pathway has been described, involving type 2 activin receptors, belonging to a family of transforming growth factor β (TGF-β) receptors.

### Endothelin Receptor Antagonists (ERAs)

Endothelin 1 (ET-1) is a potent endogenous vasoconstrictor, and its expression is increased in patients with PAH. ET-1 receptors (ET-A and ET-B) are present in endothelial and smooth muscle cells of the pulmonary arteries. ET-B receptors, predominantly expressed in endothelial cells, promote vasodilation by stimulating the production of prostacyclin and NO, in addition to increasing endothelin clearance.^26^ There are selective ET-A receptor antagonists (ambrisentan), which preserve the function of the ET-B receptor, and non-selective antagonists (bosentan and macitentan) that block both receptors. It is important to note that these molecules have a teratogenic effect and should not be used during pregnancy.

Ambrisentan is an oral endothelin receptor antagonist. The usual dose is 5 to 10 mg once daily. Two randomized, double-blind clinical trials evaluated 392 treatment-naïve patients with PAH in group 1 (95% in WHO-FC II/III) over a period of 12 weeks.^27^ There was improvement in 6MWD, WHO-FC, quality of life, time to clinical worsening, NT-proBNP values, and pulmonary hemodynamics. The most common adverse effects were lower limb edema and anemia, with reports of hepatitis in less than 3% of patients.

Bosentan, another oral ERA, is administered at an initial dose of 62.5 mg twice daily for 4 weeks, and increased to a maintenance dose of 125 mg twice daily. This drug was evaluated in six randomized clinical trials involving more than 500 patients with PAH, as well as those with PAH associated with connective tissue disease and Eisenmenger syndrome.^28^ ^-^ ^32^ There was improvement in 6MWD, WHO-FC, time to clinical worsening, and pulmonary hemodynamics. Hepatotoxicity may occur in up to 10% of cases, indicating regular monitoring of liver enzymes. There may be interaction with oral contraceptives, decreasing their therapeutic effect.^33^

Macitentan is an oral ERA used at a dose of 10 mg once daily. A double-blind, randomized clinical trial evaluated 742 patients with PAH from group 1, 97% in WHO-FC II/III, over a period of 2 years.^34^ There was a significant reduction in the composite endpoint of morbidity and mortality in patients who used the medication, regardless of prior use of other vasodilator therapy (64% were using a phosphodiesterase 5 inhibitor). The most common side effect was anemia (hemoglobin < 8 g/dL in 4.3% of patients), and no liver toxicity was reported.

### Nitric oxide pathway

NO is a soluble gas synthesized by endothelial cells and macrophages. As an intracellular and extracellular signaling molecule, it stimulates the soluble guanylate cyclase (sGC) enzyme to produce cyclic guanosine monophosphate (cGMP), which, among other effects, promotes smooth muscle relaxation, leading to vasodilation and bronchodilation. This pathway is modulated by negative feedback mediated by the degradation of cGMP by various phosphodiesterases, among which type 5 (PDE5) stands out, because it is highly expressed in the pulmonary vasculature.

Molecules that directly stimulate the sGC enzyme (e.g., riociguat) lead to the release of cGMP, both in the presence and absence of endogenous NO, causing pulmonary vasodilation. PDE5 inhibitors (PDE5i; sildenafil and tadalafil) reduce the degradation of cGMP to GMP, increasing its concentration in the endothelium with consequent arteriolar vasodilation. Common adverse effects result from the vasodilatory action (headache, facial flushing, upper respiratory congestion). PDE5i and sGC stimulators should not be used concomitantly due to the risk of severe systemic hypotension.^35^

Sildenafil is administered orally at a dose of 20 to 80 mg every 8 hours. Two related studies have evaluated its use.^36^ ^,^ ^37^ The first one was a double-blind, randomized clinical trial evaluated the use of sildenafil in more than 250 treatment-naïve patients with PAH in group 1 (96% in WHO-FC II/III), for a period of 12 weeks. There was improvement in pulmonary hemodynamics, exercise capacity and symptoms.^36^ In the open-label phase of the study, 183 patients were followed for 3 years, most using the 80 mg dose of sildenafil every 8 hours.^37^ There was a gain in exercise capacity in 60% of patients and improvement or maintenance of WHO-FC in 46% of cases. Based on these studies, regulatory agencies approved sildenafil for the treatment of PAH at a dose of 20 mg orally three times a day, up to a maximum dose of 80 mg three times a day in selected cases.^38^

Tadalafil is administered orally at a dose of 40 mg once daily. A double-blind, randomized clinical trial^39^ evaluated the use of tadalafil in 405 patients with PAH in group 1 (96% in WHO-FC II/III), half of whom were treated with bosentan, for a period of 16 weeks. There was improvement in pulmonary hemodynamics, exercise capacity, quality of life, and time to clinical worsening. In the open-label phase of the study, 213 patients were followed for a period of 10 months, with maintenance of the gain in exercise capacity.

Riociguat is used orally at an individualized dose of 0.5 to 2.5 mg every 8 hours. It was evaluated in two related studies^40^ ^,^ ^41^ A double-blind, randomized clinical trial(40) evaluated 443 patients with PAH in group 1 (95% in WHO-FC II/III), half of them treated with an ERA (44%) or a non-intravenous prostanoid (6%), for a period of 12 weeks. There was improvement in exercise capacity, pulmonary hemodynamics, NT-proBNP levels, WHO-FC, and time to clinical worsening.^40^ In the open-label phase of the study,^41^ 324 patients were evaluated for a period of 1 year, with maintenance of gains in exercise capacity, WHO-FC, and NT-proBNP levels.

Another randomized, open-label, multicenter clinical trial evaluated 211 PAH patients in group 1 and WHO-FC III, already on PDE5i therapy with or without an ERA (71% on combination therapy).^42^ Patients were either maintained on PDE5i therapy (sildenafil or tadalafil) or switched to riociguat. After 24 weeks of treatment, there was improvement in the riociguat group, with a decrease in clinical worsening events.

### Prostacyclin and prostacyclin agonists

Prostacyclin (PGI2) is a prostaglandin synthesized in endothelial cells by the action of the cyclooxygenase enzyme on arachidonic acid and is a potent vasodilator and inhibitor of platelet aggregation. This pathway is dysregulated in patients with PAH and, as a consequence, there is a decrease in cyclic adenosine monophosphate (cAMP)-a second messenger involved in vasodilatory and antiproliferative action, with a consequent reduction in its vasodilatory and antiproliferative effects.

Synthetic prostacyclins also have a vasodilatory effect, and for the treatment of PAH there are molecules administered intravenously (e.g., epoprostenol and treprostinil), subcutaneously (e.g., treprostinil), by inhalation (iloprost and treprostinil), or orally (treprostinil). There are also PGI2 receptor agonists available for oral use (selexipag). The effects of these drugs include potent vasodilation, inhibition of platelet aggregation, and antiproliferative and cytoprotective actions.^43^ Some adverse effects are common with the use of these compounds, such as headache, facial flushing, jaw pain, and diarrhea.

Epoprostenol was the first molecule specifically tested for the treatment of PAH, and the most important studies date back to the 1990s. Randomized studies versus conventional treatment conducted with a small number of patients (WHO-FC III and IV) demonstrated efficacy in idiopathic PAH through symptom improvement, increased 6MWD, and hemodynamic improvement.^25^ ^,^ ^44^ In addition to these results, the study by Barst et al. demonstrated improved mortality.^25^ Epoprostenol was also effective in patients with PAH associated with systemic sclerosis^45^ and other conditions.^46^ ^,^ ^47^ Epoprostenol has a half-life of 3-5 min, and therefore requires continuous intravenous infusion. Long-term use leads to the occurrence of adverse events associated with the infusion system, such as infection, catheter obstruction, or infusion pump malfunction. Currently, this drug is not available in the country.

Iloprost was tested via inhalation at a dose of 2.5 or 5.0 μg, 6-9 times a day (mean inhaled dose of 30 μg per day), in a randomized placebo-controlled clinical trial,^48^ which included 203 treatment-naïve patients (WHO-FC III and IV) diagnosed with PAH from group 1 or CTEPH. Treatment resulted in improvement in a combined endpoint consisting of 6MWD, WHO-FC, and clinical deterioration.^48^

Studies with subcutaneous treprostinil,^49^ inhaled treprostinil,^50^ and oral treprostinil^51^ showed improvement in 6MWD at the end of 12 weeks.

Selexipag is a selective prostacyclin receptor agonist and is available for oral use in individualized doses ranging from 200-1600 µg twice daily. A phase 3 event-driven, randomized, double-blind, placebo-controlled study included 1,156 patients with PAH who were treatment-naïve or regularly using endothelin receptor antagonists and/or phosphodiesterase type 5 inhibitors.^52^ The primary composite endpoint was the occurrence of death from any cause or any PAH-related complications by the end of patient follow-up.^52^ The risk of the composite endpoint was significantly lower in patients treated with selexipag (41.6%) versus the placebo group (27%) (RR = 0.66; p < 0.001), regardless of prior use of other pulmonary vasodilator therapy. The main adverse events were headache, diarrhea, nausea, and jaw pain.

### Inhibitor of the activin-mediated signaling pathway

Sotatercept is a novel fusion protein that combines two parts: the Fc domain of human immunoglobulin G (IgG) and the extracellular domain of the human ActRIIA receptor. Recent research has highlighted the role of altered signal transduction by members of the TGF-β superfamily, including bone morphogenetic protein receptor type II (BMPR2), activin receptor type IIA (ActRIIA), and the ActRIIA ligands activin A, activin B, growth differentiation factor 8 (GDF8), and GDF11. Sotatercept acts as a “trap” for ligands, retaining activins and growth differentiation factors involved in PAH.

The study designated STELLAR^53^ was a multicenter, double-blind, phase 3 study to evaluate subcutaneous sotatercept (initial dose: 0.3 mg per kg of body weight; target dose: 0.7 mg per kg of body weight) in adults with PAH. A total of 323 patients with stable WHO-FC II or III and receiving background therapy were randomized to receive sotatercept or a placebo every three weeks for 24 weeks. There was improvement in the primary outcome, which was 6MWD.^53^ In exploratory post hoc analysis, significant improvement was evidenced in hemodynamic parameters, such as pulmonary artery pressure, pulmonary artery compliance, right ventricular-pulmonary arterial coupling, and right heart function.^54^

A multicenter, double-blind, phase 3 study^55^ evaluated sotatercept in patients in WHO-FC-III or IV and high risk of death at 1 year according to the Registry to Evaluate Early and Long-Term PAH Disease Management Lite 2 (REVEAL Lite 2) ≥ 9 classification, who were receiving the highest tolerated dose of background pulmonary vasodilator medications. The therapeutic regimen was the same as that used in the STELLAR study.^53^ The study was terminated early based on efficacy assessed in an interim analysis, with a total of 172 patients evaluated (sotatercept group = 86 and placebo group = 86). The primary outcome, a composite of death from any cause, lung transplantation, and hospitalization for more than 24 h due to worsening of PH, occurred in 15 patients (15.1%) in the sotatercept group and in 47 patients (54.7%) in the placebo group (hazard ratio = 0.24; 95% CI: 0.13-0.43; p < 0.001). The most common adverse effects were epistaxis and telangiectasia.^56^

It is important to highlight the inequality among countries in access to treatment for PAH. The Brazilian Health Regulatory Agency approved for use drugs from all four pathways (ambrisentan, bosentan, sildenafil, riociguat, iloprost, selexipag, and sotatercept). However, only ambrisentan, bosentan, iloprost, selexipag, and sildenafil are currently available in the Brazilian Unified Health Care System. Because the basis of PAH treatment is the combination of medications, access to various therapeutic classes is fundamental.

### Lung transplantation in PAH

The perioperative management of lung transplantation in patients with PAH is complex, and mortality is still high when compared to transplantation in other diseases. There are few centers in Brazil that perform transplantation in patients with PAH, making access still difficult in our country. Therefore, the suggestions in this guideline are based on expert opinions, resulting from adaptations of international guidelines to the Brazilian reality.^57^

When to refer to a transplant center:

1. Patients who remain at non-low risk despite triple therapy;
2. Patients with veno-occlusive disease or pulmonary capillary hemangiomatosis who are not at low risk after starting treatment;
3. First episode of hemoptysis.

The timing of inclusion on the waiting list will be determined by the transplant center.

### Palliative care

Patients with PAH experience stressful and limiting symptoms such as pre-syncope, fatigue, dyspnea, and pain, which significantly impair their quality of life. Integrating palliative care approaches early in the course of the disease, along with disease-modifying therapies, can bring benefits and contribute to the overall well-being of this population, although there is still little evidence on this subject (I-C).^15^

## RISK STRATIFICATION

Risk stratification is fundamental for the therapeutic management of PAH, because it guides the intensification of treatment according to the severity of the condition. In general, it involves a multiparametric approach, combining clinical, functional, hemodynamic, and imaging variables in order to determine the 1-year mortality of patients. As a result of this assessment, patients are classified into three levels: low risk, intermediate risk, and high risk. There are several different approaches to this analysis. The first was proposed by a Swedish group^58^ and reproduced by the analysis of the European registry (Comparative, Prospective Registry of Newly Initiated Therapies for Pulmonary Hypertension-COMPERA),^59^ considering the classification of the different parameters into the three risk ranges (Table 4). According to this approach, patients receive points for each variable analyzed according to the risk range in which it is found: 1 point for low risk, 2 points for intermediate risk, and 3 points for high risk. The arithmetic mean of the values of the variables used is then calculated. Means below 1.5 classifies the patient as low risk; those between 1.5 and 2.49 classifies the patient as intermediate risk, while those greater than 2.49 classify the patient as high risk. A French study^60^ suggested an alternative approach using the same set of variables. Instead of dividing into three levels, patients are classified as low risk when all variables are within this range, otherwise, patients are classified as not low risk.

There is also a third approach, derived from the American registry (Registry to Evaluate Early and Long-Term PAH Disease Management-REVEAL).^61^ It proposes a risk calculator that performs a weighted analysis of different variables resulting in a risk score, but also allows classification into three levels. A simplified version, REVEAL Lite 2, has been validated and can also be used to classify the risk of patients with PAH.^55^

Both the analysis of the French registry and a reanalysis of the American registry also suggest a strategy based on the use of noninvasive variables for risk stratification. As a minimum assessment to be performed in the follow-up of patients with PAH, risk stratification can be based on the results of the 6MWD, serum natriuretic peptide levels, and WHO functional class. It should be emphasized, however, that a more comprehensive assessment of right ventricular function should be considered periodically, even for patients classified as low risk based solely on the minimum assessment.

More recently, risk stratification into four levels has been proposed, in order to subdivide the intermediate risk group into low-intermediate and high-intermediate.^62^ This division aims at better discrimination of severity within the group that encompasses the largest proportion of patients with PAH. Although the use of this classification is recommended by international guidelines for follow-up assessments of patients with PAH, this guideline chose to use a three-level classification for the initial assessment and a low-risk/not low-risk classification for subsequent assessments. This takes into account the available therapeutic alternatives in the country for each risk stratum.

### Treatment strategy and algorithm for PAH

**Figure 1:**
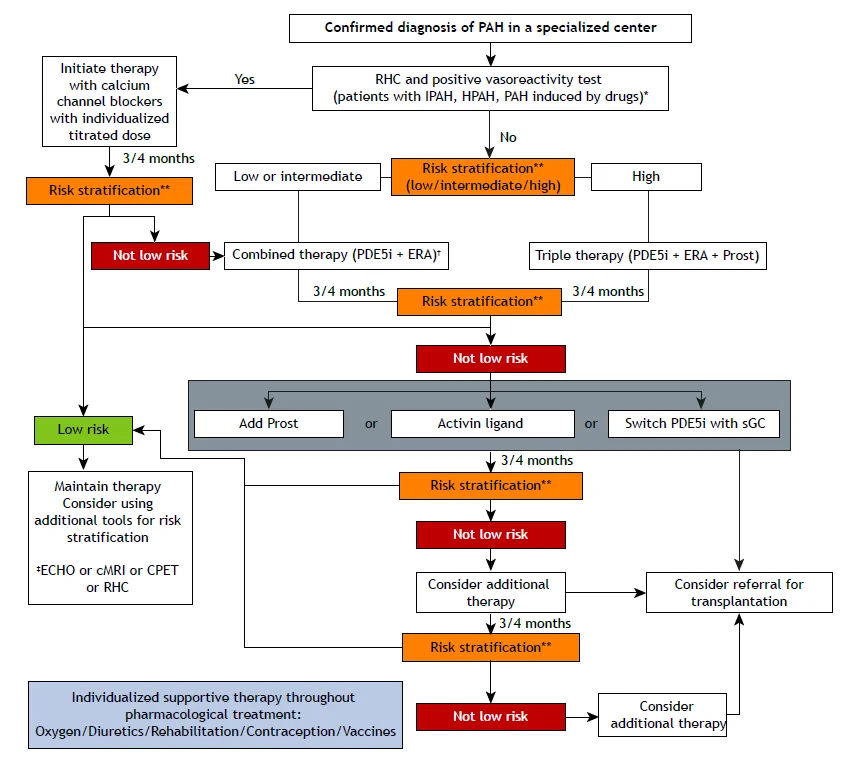
Pharmacological treatment scheme for pulmonary arterial hypertension. *If vasoreactivity test is positive, proceed with treatment using a calcium channel blocker. **Risk stratification: initial three-level assessment; during follow-up, differentiate patients who have achieved/maintained low risk from other patients. ^†^Evaluate monotherapy in selected cases, especially with cardiopulmonary comorbidities. ^‡^More comprehensive assessment of right ventricular function should be considered periodically in patients classified as low risk. PAH: pulmonary arterial hypertension; IPAH: idiopathic PAH; HPAH: hereditary PAH; RHC: right heart catheterization; PDE5i: phosphodiesterase type 5 inhibitor; ERA: endothelin receptor antagonist; Prost: prostacyclin analogue/antagonist; sGC: soluble guanylate cyclase stimulator; ECHO: transthoracic echocardiogram; cMRI: cardiac magnetic resonance imaging; CPET: cardiopulmonary exercise test; and RHC: right heart catheterization.

The treatment of PAH aims to reduce morbidity, improve quality of life, and prolong patient survival. Taking into account risk stratification, the goal of treatment is always to achieve a low-risk profile. For this purpose, using a combination of drugs has become an essential part of the management strategy for patients with PAH (Figure 1). For the development of this algorithm, some guiding questions were considered to assess the robustness of the existing evidence (PICO questions 1 and 2). Furthermore, risk stratification was used, both in the initial assessment and in reassessments every 3-4 months, as the basis for therapeutic decisions to be adopted.

## PICO QUESTIONS

Question 1: Should we recommend initial dual combination therapy compared to monotherapy for patients with PAH?

### Evidence

Using defined criteria for inclusion of studies with PAH patients, combinations of pulmonary vasodilator drugs acting on nitric oxide pathways (sildenafil, tadalafil, riociguat), prostacyclin (epoprostenol, treprostinil, iloprost, selexipag), and endothelin receptor antagonists (macitentan, bosentan, ambrisentan) were considered. The search resulted in 1,605 articles; after eliminating duplicates, 562 articles remained, of which 452 were excluded for not belonging to the scope of the question, according to the title and abstract screening. Of the remaining 110, only 4 studies passed for joint evaluation with experts. The main reasons for exclusion were: inadequate interventions, lack of randomization, phase I studies, ineligible population, lack of drug association, observational designs, duplicate studies or studies without results, and use of unapproved drugs. Of the four studies, two were excluded because they evaluated combinations of three vasodilators, and one of the studies was retrospective. One study was excluded because it included patients with CTEPH (14%).

Only one randomized clinical trial^63^ was included, comparing initial combination therapy (ambrisentan, 10 mg/day + tadalafil, 40 mg/day; n = 253) versus monotherapy (ambrisentan, n = 126; tadalafil, n = 121), in patients with PAH in WHO-FC II or III. The combined primary outcome (clinical worsening, composite of death, hospitalization due to PAH worsening, disease progression, or poor long-term clinical response) was favorable to the combination treatment, 46 patients (18%) vs. the monotherapy groups-ambrisentan, 43 patients (34%), and tadalafil, 34 patients (28%). The hazard ratio was 0.5 (95% CI: 0.35-0.72; p < 0.001), considering patients treated with monotherapy in a pooled manner. There was a significant increase in 6MWD, with a median of 48.98 m (25th-75th percentiles: 4.63 m to 85.75 m), in the combined initial treatment group, when compared to monotherapy, assessed in a pooled manner (23.8 m; 12.25 m to 64.53 m, p < 0.001). There was also a greater reduction in NT-proBNP levels (geometric mean of −67.2 pg/dL vs. −50.4 pg/dL; p < 0.001). No significant difference was observed in the variation of WHO-FC between the groups.^63^

### Recommendation

For patients with PAH, we recommend initial combination therapy (based on the study that evaluated the combination of ambrisentan and tadalafil) in comparison with monotherapy (strong recommendation, high quality of evidence).

### Comments

Initial treatment of PAH in patients who do not respond to vasodilator testing often involves dual oral therapy, combining a PDE5i, such as sildenafil, and an ERA, such as ambrisentan or bosentan. This approach aims to improve pulmonary vasodilation, functional capacity, and hemodynamic parameters. Although the evidence for dual therapy is fundamentally based on one study^63^ that used ambrisentan and tadalafil, the proposal of this treatment guideline expands the concept of combined treatment to other drugs in the PDE5i and ERA classes available in the country.

In patients who do not achieve a low-risk profile with dual therapy, it is possible to escalate treatment to sequential triple therapy with the addition of a prostacyclin. In this context, oral selexipag (level of evidence I-B) or inhaled iloprost (level of evidence IIa-C) can be added to the previously mentioned agents in order to improve cardiac output. Sequential triple therapy with prostanoids is supported by studies and is indicated in cases of suboptimal response to dual therapy.^52^ ^,^ ^64^ ^,^ ^65^

An alternative to sequential triple therapy with prostanoids, in cases of patients who are not at low risk, is the addition of an activin pathway drug (sotatercept; evidence I-A).^54^ ^,^ ^56^

Question 2: Should we recommend switching between drugs of the same class for patients with PAH using combination therapy and who are classified as having intermediate risk?

### Evidence

Following a previously defined methodology, we selected studies that included patients classified as group 1 for PH and that evaluated substitutions between pulmonary vasodilator drugs within their respective pathways: NO (replacing sildenafil/tadalafil with riociguat), PGI2 (treprostinil, iloprost, or selexipag with epoprostenol), and ERA (macitentan, bosentan, or ambrisentan among themselves). The initial search identified 2,370 articles; 1,054 were excluded due to duplication. After screening by title and abstract, 1,286 were eliminated due to inadequacy to the scope. Of the 30 articles evaluated in full, only 1 was included in the qualitative synthesis. Exclusions occurred due to: lack of randomization, case series, inappropriate interventions, duplicate populations, study protocols, reviews, retrospective designs, or exclusive use of monotherapy.

That study was an open-label, non-blinded trial that randomized patients with PAH on combined oral use of an endothelin receptor antagonist and an PDE5i. The intervention group switched from the PDE5i (sildenafil ≥ 60 mg/day or tadalafil 20-40 mg/day) to riociguat (up to 2.5 mg every 8 hours, as tolerated; n = 111), while the control group maintained their previous treatment. The primary outcome was presenting with clinical improvement at 24 weeks (defined as improvement in at least two of the following parameters and without worsening: 6MWD, NT-proBNP and WHO-FC). This outcome was achieved in 41% of patients in the riociguat group vs. 20% in the control group (OR = 2.78; 95% CI: 1.53-5.06; p = 0.007). Clinical worsening occurred in 1% of the riociguat group vs. 9% of the control group (OR = 0.10; 95% CI: 0.01-0.73; p = 0.0047). The most common adverse effects in the riociguat group included hypotension (14%), headache (13%), and dyspnea (9%).^42^

### Recommendation

For PAH patients using combination therapy and classified as having intermediate risk, we suggest replacing sildenafil/tadalafil with riociguat (conditional recommendation, very low quality of evidence).

### Comments

Switching from PDE5i to riociguat may be an option for patients who have not achieved low risk after initiating treatment with dual oral therapy, or for patients who have not tolerated the adverse effects related to prostacyclin-based medications. Considering the few therapeutic alternatives in the country for patients on triple therapy who have not achieved low risk, switching from PDE5i to riociguat could be considered in these cases, although this population has not been specifically studied.

## TREATMENT OF PH-GROUP 2

Question 3: Should pulmonary vasodilator therapy be used to treat PH due to left heart disease (PH Group 2) in comparison with the clinical management of the underlying disease?

### Evidence

Using the described methodology, criteria for the inclusion of articles were defined, including diseases affecting the left heart chambers and valves, and the pulmonary vasodilator drugs to be evaluated for their treatment (sildenafil, tadalafil, riociguat, bosentan, ambrisentan, macitentan, selexipag, iloprost, epoprostenol, and treprostinil). The initial search identified 13,632 articles. After screening by title and abstract, 13,315 studies were excluded for not meeting the criteria of design, population, or intervention. Of the 317 articles evaluated in full, 307 were excluded for the same reasons, resulting in 10 studies included in the qualitative synthesis. Meta-analysis was not feasible due to the heterogeneity among the studies, both in relation to the diseases addressed and the therapies used.

### PDE5i

Two studies evaluated the treatment of patients in group II, who had heart failure with reduced ejection fraction (HFrEF). Cooper et al.^66^ evaluated patients with heart failure in functional class II-III (WHO), pulmonary artery systolic pressure (PASP) ≥ 40 mmHg and left ventricular ejection fraction (LVEF) ≤ 40%, assessed by echocardiography. Patients were treated with sildenafil 40 mg every 8 hours (n = 45) or placebo (n = 24) for two weeks. The primary outcome of the 6MWD showed no statistically significant difference at the 8-week [estimated difference: 11.2 (95% CI: −13.4 to 35.8; p = 0.37)] and 24-week [estimated difference: −2.8 (95% CI: −38.5 to 32.9; p = 0.88)] assessments. Quality of life outcomes assessed by visual scales and specific questionnaires also showed no significant difference.^66^ In the study by Lewis et al.,^67^ patients with LVEF ≤ 40%, WHO-FC II-IV, and mPAP > 25 mmHg were evaluated. Patients were treated with sildenafil (n = 17) or placebo (n = 17) for 12 weeks. The sildenafil dose was increased every two weeks, from 25 to 75 mg every 8 h. There was significant improvement in the primary outcome, VO_2_ at peak exercise, favoring the group that used sildenafil (1.8 ± 0.7 mL • kg-1 • min-1) vs placebo (−0.27 mL • kg-1 • min-1; p =0.02). Cardiac index, systolic volume (SV), and PVR, assessed during exercise, also improved significantly in the treatment group. Quality of life, assessed by a specific questionnaire, WHO-FC and 6MWD, was superior in the group that used sildenafil at six months of follow-up. There was no statistical difference between the groups regarding serious adverse events.^67^

Two studies evaluated treatment with sildenafil 60 mg every 8 h versus placebo for 12 weeks in patients with heart failure and preserved ejection fraction (HFpEF) and hemodynamic criteria for pulmonary hypertension.^68^ ^,^ ^69^ Both studies included the same number of patients (n = 52, 1:1 distribution) and assessed the response to exercise in cardiopulmonary exercise testing. There was no statistically significant difference in variables considered relevant in both studies, such as peak VO_2_.^68^ ^,^ ^69^ There was no significant improvement in quality of life between the groups (Kansas City Cardiomyopathy Questionnaire).^69^ A third study evaluated sildenafil at a dose of 50 mg every 8 h (n = 22) versus placebo (n = 22), with measurements of baseline, 6-week, and 12-month pulmonary hemodynamics in patients with HFpEF. There was a significant reduction in PVR and PAOP and an increase in cardiac index at 6 and 12 months in the group that used sildenafil, compared to placebo and when compared to their own baseline values. There was an improvement in quality of life, but the study did not specify the questionnaire used.^70^

The use of sildenafil was evaluated in patients undergoing left heart valve replacement or repair and PH defined by catheterization with a mPAP ≥ 30 mmHg. Patients treated with sildenafil at a dose of 40 mg every 8 h (n = 104) were compared with a placebo group (n = 96). The primary outcome combined death, hospitalization for heart failure, change in WHO-FC score, and global self-assessment after 24 weeks, and was favorable to patients receiving placebo [OR: 0.39; 95% CI: 0.22-0.67; p < 0.001]. The estimated 6-month survival without clinical complications of death or hospitalization for heart failure was favorable to the placebo group (hazard ratio: 2.0; 95% CI: 1.0-4.0; p = 0.044). There was no statistical difference in the 6MWD or in the OMS-FC.^71^

#### Recommendation

For patients with PH resulting from left heart disease, we suggest not using sildenafil (conditional recommendation, very low quality of evidence).

### Soluble guanylate cyclase (sGC) stimulator

Patients with HFrEF, defined by echocardiogram and pulmonary hemodynamic assessment, were treated with riociguat using three different doses, three times daily: 0.5 mg (n = 32), 1 mg (n = 33), and 2 mg (n = 67) vs. placebo (n = 69) in a phase II study. Patients were treated for 16 weeks. The primary outcome of PAPm reduction showed no statistically significant difference. There was a significant reduction in PVR (−239.3 dynes•s^−1^•cm^−5^; 95% CI, -363.4 to -115.3; p =0.0002) and an increase in cardiac index (0.4 L•min^−1^•m^−2^; 95% CI, 0.2-0.5; p = 0.0001) and SV (5.2 mL•m^−2^; 95% CI, 2.0-8.4; p = 0.0018), favorable to patients who used 2 mg of riociguat every 8 h. There was a significant improvement in quality of life, assessed by the Minnesota Living With Heart Failure Questionnaire, favorable to the riociguat group.^72^

Dachs et al. evaluated the use of riociguat (n = 58) vs. placebo (n = 56) in patients with HFpEF defined by echocardiographic and hemodynamic criteria, in a phase II study.^73^ Patients who received riociguat had their dose titrated from 0.5 to 1.5 mg, three times a day. Patients were evaluated at the end of 26 weeks of treatment. The primary outcome of increased cardiac output was favorable to the group that used riociguate (least squares difference: 0.54 L/min, 95% CI: 0.112-0.971; p = 0.0142), and PVR also decreased significantly in these patients. A greater number of patients presented with hypotension in the riociguate group.^73^

#### Recommendation

We do not have sufficient evidence to recommend or not recommend the use of riociguat in patients with PH resulting from left heart disease (conditional recommendation, moderate quality of evidence).

### Endothelin receptor antagonists

Treatment with bosentan in patients with HFpEF, defined by echocardiographic and hemodynamic criteria, was evaluated in a pilot study.^74^ Nine patients received bosentan vs. placebo (n = 11). There was no statistically significant difference between the groups in the 6MWD: treatment group = from 309.7 ± 96.3 m (baseline) to 317.0 ± 126.1 m (week 12) and to 307.0 ± 84.4 m (week 24;h; p = 0.86); placebo group = 328.8 ± 79.6 m (baseline), 361.6 ± 98.2 m (week 12), 384.0 ± 74.9 m (week 24;, p = 0.075). There was no significant change in functional class or quality of life as assessed by the Medical Outcomes Study 36-item Short-Form Health Survey and the Minnesota Living with Heart Failure Questionnaire.^74^

#### Recommendation

For patients with PH due to left heart disease, we suggest not using bosentan (conditional recommendation, low quality of evidence).

One study evaluated treatment with macitentan for patients with PH associated with left heart disease with a pre- and post-capillary component (diastolic pressure gradient ≥ 7 mmHg PVR > 3 WU).^75^ Patients received macitentan 10 mg (n = 31) vs. placebo (n = 32) for 12 weeks. The primary composite outcome of fluid retention and worsening functional class (NYHA) showed no statistically significant difference between the groups, nor did the hemodynamic variables of PVR, right atrial pressure, and cardiac index. Numerically, more patients in the macitentan group experienced fluid retention compared to the control group.^75^

#### Recommendation

For patients with PH due to left heart disease, we recommend against using macitentan (strong recommendation, moderate quality of evidence).

### Comments

PH secondary to left heart disease (group 2) includes HFpEF or HFrEF, valvular heart disease, and acquired or congenital heart disease that leads to post-capillary pattern of PH.^76^ It is the most common form of PH in clinical practice, accounting for 65-80% of cases.^77^ The prevalence of PH varies among different heart diseases, being evidenced in about 70% of HFrEF, between 36% and 83% of HFpEF, and 15-60% in valvular diseases.^78^ The presence of PH secondary to left heart disease is associated with worse prognosis and higher mortality.^79^ The pathophysiology is complex and involves passive upstream transmission of elevated pressure in the left chambers, endothelial dysfunction, pulmonary vascular remodeling with increased PVR, RV dysfunction, functional tricuspid regurgitation, and uncoupling between the RV and the pulmonary arterial bed.^77^ ^,^ ^80^

PH resulting from left heart disease is a challenge since the diagnosis, because some patients do not have the classic hemodynamic pattern of isolated post-capillary PH and may present with combined pre- and post-capillary features.^81^ Invasive evaluation is essential when the results imply a change in management, such as in the context of evaluation for heart transplantation, when pulmonary vascular reactivity tests may be necessary.^82^ ^,^ ^83^

The initial management of PH secondary to left heart disease consists of optimizing the treatment of the underlying heart disease, with an individualized phenotypic approach.^81^ This strategy includes general measures such as cardiopulmonary rehabilitation, pharmacological therapies for heart failure, and endovascular or surgical interventions in valvular heart disease. Clinical trials with targeted drugs for PH associated with left heart disease have shown frustrating results. Although some studies have shown hemodynamic and functional improvements, there has been no consistent benefit in hard clinical outcomes, and, in some cases, potential harm has been observed.^82^

We do not recommend the routine use of specific therapies in patients with PH resulting from left heart disease.

Question 4: Should pulmonary vasodilator therapy be added to the clinical management of the underlying respiratory disease in patients with group 3 PH?

### Evidence

In accordance with the described methodology, inclusion criteria were established focusing on respiratory diseases associated with hypoxemia, with an emphasis on the evaluation of pulmonary vasodilator agents (sildenafil, tadalafil, riociguat, bosentan, ambrisentan, macitentan, selexipag, iloprost, epoprostenol, and treprostinil). The initial bibliographic search identified 16,395 articles, of which 16,048 were excluded after screening by title and abstract for not meeting the design, population, or intervention criteria. Of the 347 articles evaluated in full, 340 were excluded for the same reasons, in addition to 5 studies that were duplicates, resulting in 7 studies included in the qualitative synthesis. Meta-analysis was not possible due to the heterogeneity of the studies, particularly regarding the types of respiratory diseases investigated and the vasodilator therapies employed.

### PDE5i

Three studies evaluated vasodilators related to the NO pathway. Two studies evaluated tadalafil vs. placebo for the treatment of patients with COPD. Maron et al.^84^ considered invasive hemodynamic criteria for defining pre-capillary PH and including patients. Fourteen patients were studied in the placebo group and 28 patients in the tadalafil 40 mg/day group. The outcome of 6MWD gain at 12 months showed no significant difference between patients treated with tadalafil vs. placebo (2 1 m; 95% CI: −9 to 40 vs. 17 m; 95% CI: −1.7 to 49); p = 0.65). There was no difference between the groups in hemodynamic variables and in the assessment of quality of life by specific questionnaires after 6 months of treatment. Patients who used tadalafil experienced fewer exacerbations and improved dyspnea, as measured by the University of California San Diego Shortness of Breath Questionnaire.^84^ Goudie et al.^85^ used echocardiographic parameters to define suspected PH. Sixty patients received tadalafil 10 mg/day and 60 patients received placebo. There was no difference between the groups in the primary outcome of 6MWD after 12 weeks (0.5 m; 95% CI: −11.6 to 12.5; p = 0.937). There was no significant difference in the assessment of quality of life.^85^

A study evaluated sildenafil 20 mg every 8 hours (n = 18) vs. placebo (n = 10) for 16 weeks in patients with COPD and hemodynamic criteria for pre-capillary PH.^86^ There was a statistically significant improvement in hemodynamic parameters: mean difference in PVR between sildenafil and placebo groups = −1.38 WU (95% CI ≤ −0.05 WU; p = 0.044), in indexed stroke volume = 7.6 mL/m^2^ (95% CI: ≥ 3.7 mL/m^2^), and in cardiac index of 0.4 L/min/m^2^ (95% CI: ≥ 0.2 L/min/m^2^; p = 0.004). Regarding secondary outcomes, significant improvements were observed in BMI, airflow obstruction (BODE), and DL_CO_%. There was a trend toward improvement of 6MWD in patients who used sildenafil.^86^

#### Recommendation

There is insufficient evidence to recommend or not recommend the use of PDEi in conjunction with clinical management of the underlying disease in patients with COPD and PH (group 3; conditional recommendation, poor quality of evidence).

### Soluble guanylate cyclase (sGC) stimulator

A multicenter study evaluated riociguat for the treatment of patients diagnosed with idiopathic pulmonary fibrosis (IPF), with pre-capillary PH confirmed by RHC, who had PASP ≥ 95 mmHg.(87) The riociguat dose was titrated over 10 weeks. In the subsequent 16 weeks, patients (n = 73) used the dose achieved at titration, from 0.5 mg to 2.5 mg every 8 h. Patients in the placebo group (n = 74) were treated for 26 weeks. The study was prematurely terminated due to an increase in serious adverse events and mortality in patients receiving riociguat. The primary outcome of the 6MWD showed no statistically significant difference between the groups (least squares mean difference = 21 m; 95% CI: -9 to 52).^87^

#### Recommendation

We recommend against using riociguat in conjunction with clinical management of the underlying disease for patients with IPF and PH (group 3; strong recommendation, moderate quality of evidence);

### Endothelin receptor antagonists

The study by Corte et al.^88^ evaluated treatment with bosentan in patients with fibrosing interstitial lung disease (IPF, nonspecific interstitial lung disease, or chronic hypersensitivity pneumonitis). The diagnosis of pre-capillary PH was established by RHC and patients with mPAP ≥ 25 mmHg and PAP ≤ 15 mmHg were included. Of the 60 patients recruited in the study, only hemodynamic data from 39 patients were available at the end of the study: bosentan (n = 25) and placebo (n = 14) groups. The primary outcome of a 20% reduction in PVR index was negative (p = 0.97). There was no statistical difference in the analysis of the other hemodynamic parameters evaluated: variation in 6MWD, DL_CO_, FVC, and quality of life, assessed by the Cambridge Pulmonary Hypertension Outcome Review questionnaire.^88^

#### Recommendation

We suggest not using bosentan in conjunction with clinical management of the underlying disease for patients with fibrosing lung disease and PH (group 3; conditional recommendation, moderate quality of evidence).

Soltz et al.^89^ evaluated the use of bosentan (n = 20) vs. placebo (n = 10) in patients with COPD. The criterion for PH was echocardiographic (PASP ≥ 30 mmHg). Six patients in the placebo group and 14 patients in the bosentan group presented with PH according to the adopted criterion. There was no statistically significant difference in the 6MWD (p = 0.474). The secondary outcomes of quality of life, pulmonary function, and right ventricular function on echocardiography showed no statistically significant differences.^89^

#### Recommendation

We suggest not using bosentan in conjunction with clinical management of the underlying disease for patients with COPD and PH (group 3; conditional recommendation, moderate quality of evidence).

### Prostacyclin analogues

Waxman et al. evaluated the use of inhaled treprostinil for the treatment of patients with diffuse interstitial lung disease and precapillary PH, defined by invasive hemodynamics. Patients in the treprostinil group (n = 163) titrated the dose until reaching a maximum of 12 breaths of the nebulized solution (0.6 mg/mL), four times a day. Patients in the placebo group performed inhalations in a similar way. After 16 weeks, there was a significant increase in the primary outcome of 6MWD (31.12 m; 95% CI: 16.85-45.39; p < 0.001). The secondary outcomes of clinical worsening and NT-proBNP levels were also statistically favorable to the treprostinil group.^90^

#### Recommendation

We suggest the use of inhaled treprostinil associated with the clinical management of the underlying disease for patients with interstitial lung disease and PH (conditional recommendation, moderate quality of evidence).

#### Comments

PH associated with chronic lung diseases and hypoxemia, group 3, is the second most common type of PH and causes significant morbidity and mortality.^91^ The heterogeneity of the pathogenic mechanisms and the specific clinical presentations of each underlying disease make it difficult to assess the effectiveness of the different treatments.^92^ In general, comparing patients with PH in group 3 with individuals in the other PH groups, it is noted that the former are older, have a more severe initial clinical presentation, and have lower survival rates than patients with PAH.^93^ Treatment is based on optimizing underlying pulmonary conditions, controlling comorbidities and supportive treatment, such as home oxygen therapy, non-invasive ventilation and pulmonary rehabilitation. The subgroup of patients in PH group 3 and presenting with greater hemodynamic compromise and PVR greater than 5 WU should be evaluated in centers specialized in pulmonary circulation.^15^ ^,^ ^91^ ^,^ ^92^

A study demonstrated that the use of ambrisentan was associated with an increased risk of clinical worsening in patients with interstitial lung disease with and without PH, a finding that supports the contraindication of this molecule in this subgroup of patients.^94^ Riociguat was associated with an increased risk of clinical worsening, including high mortality in patients with PH and IPF,^87^ so we recommend not using this drug for these patients.

Among the available medications, inhaled treprostinil seems promising for the treatment of PH due to interstitial lung disease, but the same effect has not been observed in patients with PH associated with COPD. The study called PERFECT, published after the systematic review of this guideline, was interrupted prematurely due to evidence that inhaled treprostinil increased the number of adverse events in individuals with COPD and PH. Therefore, the use of this medication in an inhaled form is not recommended for patients diagnosed with COPD (III-B).^90^ ^,^ ^95^ ^,^ ^96^

For patients with COPD and PH, there is no evidence to recommend phosphodiesterase inhibitors, and we suggest not using bosentan as drugs in association with the clinical management of the underlying disease.

Recommendations related to PICO questions are summarized in Chart 5.

**Chart 4:** Pulmonary arterial hypertension risk stratification.

Risk and estimated 1-year mortality	Low risk < 5%	Intermediate risk 5-20%	High risk > 20%
Modifiable variables			
Signs of RHF	Absent	Absent	Present
Symptom progression	No	Slow	Fast
Syncope	No	Occasionally	Repeated
WHO-FC	I, II	III	IV
6MWD	> 440 m	165-440 m	< 165 m
Cardiopulmonary exercise test	VO_2_peak >15 mL/min/kg (> 65% predicted) VE/VCO_2_ slope < 36	VO_2_peak 11-15 mL/min/kg (35-65% preditcted VE/VCO_2_ slope 36-44	VO_2_peak < 11 mL/min/kg (<35% predicte) VE/VCO_2_ slope ≥ 45
Biomarkers: BNP and NT-proBNP	BNP < 50 ng/L NT-proBNP < 300 ng/mL	BNP 50-800 ng/L NT-proBNP 300-1100 ng/mL	BNP > 800 ng/L NT-proBNP > 1100 ng/mL
Echocardiography	RA area < 18 cm^2^ TAPSE / PASP > 0,32 mm/mmHg No pericardial effusion	RA area 18-26 cm^2^ TAPSE / PASP 0,19 -0,32 mm/mmHg Minimal pericardial effusion	RA area >26 cm^2^ TAPSE / PASP < 0,19 mm/mmHg Pericardial effusion
cMRI	RVEF > 54% SVI >4 0 mL/m^2^ RVESVI < 42mL/m^2^	RVEF 37-54% SVI 26-40 mL/m^2^ RVESVI 42-54 mL/m^2^	RVEF < 37% SVI < 26 mL/m^2^ RVESVI > 54 mL/m^2^
Hemodynamics	RAP < 8 mmHg CI > 2,5 L/min/m^2^ SVI > 38 mL/m^2^ SvO_2_ > 65%	RAP 8-14 mmHg CI 2-2,4 L/min/m^2^ SVI 31-38 mL/m^2^ SvO_2_ 60-65%	RAP > 14 mmHg CI < 2 L/min/m^2^ SVI < 31 mL/m^2^ SvO_2_ < 60%

RHF: right heart failure; WHO-FC: WHO functional class; VO_2_: oxygen intake; V_E_/VCO_2_: Ventilatory equivalent for carbon dioxide; 6MWD: six-minute walk distance; RA: right atrium; TAPSE: tricuspid annular plane systolic excursion; PASP: pulmonary artery systolic pressure; cMRI: cardiac magnetic resonance imaging; RVEF: right ventricular ejection fraction; SVI: systolic volume index; RVESVI: right ventricular end-systolic volume index; RAP: right atrial pressure; CI: cardiac index; SvO_2_: mixed venous oxygen saturation.

**Chart 5:** Summary of recommendations related to PICO questions.

Questions		Recommendations	Recommendation level	Quality of evidence
Question 1 - Should we recommend initial dual combination therapy compared to monotherapy for patients with PAH?		For patients with PAH, we recommend initial combination therapy (based on the study that evaluated the combination of ambrisentan and tadalafil) in comparison with monotherapy (strong recommendation, high quality of evidence).	Strong	High quality of evidence
Question 2: Should we recommend switching between drugs of the same class for patients with PAH using combination therapy, classified as intermediate risk?		For PAH patients on combination therapy classified as intermediate risk, we suggest replacing sildenafil/tadalafil with riociguat.	Conditional	Very low quality of evidence
Question 3: Should pulmonary vasodilator therapy be used for the treatment of PAH resulting from left heart disease (group 2 PAH) compared to clinical management of the underlying disease?	*Phosphodiesterase 5 inhibitors*	For patients with PAH due to left heart disease, we suggest not using sildenafil.	Conditional	Very low quality of evidence
	*Soluble guanylate cyclase (sGC) stimulator*	There is insufficient evidence to recommend or not recommend the use of riociguat in patients with PAH due to left heart disease.	Conditional	Moderate quality of evidence
	*Endothelin receptor antagonists*	For patients with PAH due to left heart disease, we suggest not using bosentan.	Conditional	Low quality of evidence
		For patients with PH resulting from left heart disease, we recommend not using macitentan.	Strong	Moderate quality of evidence
Question 4: Should pulmonary vasodilator therapy be associated with clinical management of the underlying respiratory disease in patients with group 3 (PH).	*Phosphodiesterase 5 inhibitors*	There is insufficient evidence to recommend or not recommend the use of phosphodiesterase inhibitors associated with clinical management of the underlying disease in patients with COPD and PH (group 3).	Conditional	Poor quality of evidence
	*Soluble guanylate cyclase (sGC) stimulator*	We recommend not using riociguat associated with clinical management of the underlying disease for patients with IPF and PH (group 3).	Strong	Moderate quality of evidence
	*Endothelin receptor antagonists*	We suggest not using bosentan associated with clinical management of the underlying disease for patients with fibrosing lung disease and PH (group 3).	Conditional	Moderate quality of evidence
	*Prostacyclin analogs*	We suggest the use of inhaled treprostinil associated with clinical management of the underlying disease for patients with ILD and PH.	Conditional	Moderate quality of evidence

PAH: pulmonary arterial hypertension; PH: pulmonary hypertension; sGC: soluble guanylate cyclase; IPF: idiopathic pulmonary fibrosis; and ILD: interstitial lung disease.

### Treatment in Group 4 PH (chronic thromboembolic PH)

Group 4 PH, also called chronic thromboembolic PH (CTEPH), occurs primarily due to the presence of chronic thromboembolic material obstructing the pulmonary arteries, which causes an increase in pressure in these arteries and right ventricular overload. In addition to the process of vascular occlusion, there is the phenomenon of vascular remodeling both in the occluded regions and in the more preserved regions.^97^

CTEPH is the only potentially curable form of pulmonary hypertension, as in many cases pulmonary thromboendarterectomy (PEA) surgery can remove chronic thrombi, relieving the obstruction.^98^ However, the recommended therapeutic approach is multimodal and may include, in addition to surgical treatment, balloon pulmonary angioplasty and the use of specific drugs. For a more in-depth approach to the topic, the Brazilian guideline on chronic thromboembolism can be consulted.^97^

All patients with CTEPH require prolonged anticoagulation to prevent new thromboembolic events. Both direct oral anticoagulants and warfarin can be used, although the preferential use of warfarin has been suggested after some discussions in the literature.^99^ ^,^ ^100^ Patients diagnosed with CTEPH and antiphospholipid antibody syndrome should not be anticoagulated with direct oral anticoagulants.^101^ Patients should be evaluated regarding the indication of oxygen therapy and diuretics to manage symptoms and control right heart failure.

PEA is the treatment of choice for patients with operable CTEPH. In this procedure, organized thrombi from the pulmonary arteries are removed, leading to a potential cure of PH in these individuals in the majority of cases, in addition to causing a significant improvement in survival and quality of life.^98^

In patients considered inoperable or who refuse surgery, balloon pulmonary angioplasty may be an alternative, offering a less invasive option than PEA and with good results in selected cases.^102^

For patients with inoperable CTEPH or who remain with residual PH after PEA, specific targeted drug treatment has also been shown to be effective. Riociguat is approved and proven to improve hemodynamics and functional capacity, as evidenced in one study.^103^ However, this drug is not currently available in the health care public system in Brazil.

Integrated or combined treatment, involving surgery, riociguat, anticoagulation, angioplasty, and clinical support, provides the best chances of controlling the disease and improving the quality of life for patients with CTEPH.

## GROUP 5

Group 5 in the PH classification involves diseases with multifactorial or unknown mechanisms, which may be hematological diseases (hereditary or acquired hemolytic anemias and myeloproliferative diseases), systemic diseases (sarcoidosis, pulmonary Langerhans cell histiocytosis, neurofibromatosis type 1), metabolic diseases (glycogenoses, Gaucher disease), chronic renal failure (with or without hemodialysis), pulmonary tumor thrombotic microangiopathy, mediastinal fibrosis, and complex congenital heart diseases (Chart 1).^1^ This is a very heterogeneous group, with different pathophysiological mechanisms, the evidence of which is mostly based on observational studies, although some conditions contribute to a considerable number of cases, such as hemolytic anemia (Table S8).^104^ ^,^ ^105^

The investigation of PH in group 5 patients may require confirmation of PH, in addition to defining the hemodynamic pattern by RHC.^15^ The initial stage of treatment is focused on the underlying disease.^106^ We recommend that patients with group 5 PH be referred to specialized centers.
